# Domestic Correspondence

**Published:** 1884-06

**Authors:** 


### Domestic Correspondence.

---

[Having now with some patience published in full the statements and counter-statements of both parties to the question considered in the subjoined communication, we must decline to receive any further rejoinder from either side, as the subject is not specially interesting to the readers of this journal.]—EDS.

DUNNING, ILL., APRIL 21, 1884.

TO THE EDITORS OF THE CHICAGO MEDICAL JOURNAL AND EXAMINER.

*Gentlemen:*—In the January number of your journal was published my article on Practice at the County Infirmary. Among other cases reported was that of J. C. McMullen, who, in trying to alight from a freight train while in motion, Nov. 1, 1882, sustained a compound comminuted fracture of the tibia and fibula, necessitating an amputation of the upper portion of the middle third of his left leg. The operation was performed by Dr. D. B. Fonda, who, in his reply to my article in the April number of your journal, takes exception to that portion of the article alluding to the case. First, because he considers himself persecuted on account of his close proximity to this and neighboring public institutions. Second, that the date the patient was admitted to this institution is erroneously stated in my article. Third, because of the statement that no ligature ends were found outside the stump when the patient was admitted, but that a "large bundle" was discovered where the stump sloughed. In reply, I disclaim all jealousy of or desire to injure Dr. F. or any other neighboring practitioner. If there is any feeling existing, a comparison between the two replies will show where it is. The remaining points are covered by affidavits by Dr. Morgan, of Chicago; Dr. Bennett, of Minneapolis; Alexander Bruce, of this institution, and myself. As Dr. F. has seen fit to introduce several affidavits in his reply by himself, his son-in-law, and a reputed medical student, I consider myself extremely fortunate in having the affidavits of several medical gentlemen who are acquainted with some of the facts in the case. A. W. HAGENBACH, M.D.

I, A. W. Hagenbach, being duly sworn, on my oath do say, that I have in my possession the first copy of the article on "Practice at the County Infirmary," published in the January number of the CHICAGO MED. JOUR. AND EXAMINER, and that the first two dates in the case of "Ligature of Femoral and External Iliac Arteries" are there given as Nov. 1 and Nov. 4, instead of Oct. 22 and 26, and that the dates were taken from the Male Hospital Register of this institution. I further say that when J. C. McMullen, the person alluded to in my article, was admitted to the infirmary after the amputation of upper portion of the middle third of his left leg, there were no ligature ends outside of the stump, and that as the stump opened up by sloughing, numerous ligatures were found at the bottom of the wound, some cut short, others long and matted together. Further, that the main facts of the article are taken from notes made at the time by Dr. Morgan, of the Cook County Hospital, and myself, and that the case as reported is to the best of my knowledge in every respect true and correct.

A. W. HAGENBACH, M.D.,
Med. Supt. Cook Co. Infirmary.

Dr. Allen W. Hagenbach, personally known to me, has been duly sworn to the fact stated and subscribed to in the foregoing instrument, this eighteenth day of April, A.D., 1884.

[SEAL.] G. T. WOLF, Notary Public.
Dunning, Illinois.

---

CHICAGO, APRIL, 19, 1884.

EDITORS OF THE CHICAGO MEDICAL JOURNAL AND EXAMINER.

*Gentlemen:* — In reply to Dr. Blackburn's *a priori* criticism I beg leave to state: First, Dr. Worcester (op. cit., p. 274) says, "there are two or three agents employed by the old school whose use you will do well to bear in mind, both for your patients' sake and *because you will not want to see your patient pass into another physician's hands.*" The agents alluded to are: chloral hydrate, in thirty grain doses; potassium bromide in ten grain doses; morphine in half-grain doses; hyoscyamine in three-quarter grain doses. It may be honest for Dr. Blackburn to practice in this way, but to me it seems the worst sort of therapeutic dishonesty. Second, Dr. Worcester did graduate from a regular school, practiced *medicine* for several years, then adopted a sectarian designation, and became a professor in a sectarian school. Such a change of views, if honest, is to be respected, but the honesty of such a course is usually suspected where a man profits by a change of views, and at the same time advocates the procedures cited from Dr. Worcester. If Dr. Blackburn's statement, that my assertion of the facts of Dr. Worcester's career above is false, be then true, Dr. Worcester committed perjury at the time of the Guiteau trial, for he swore to the facts above stated, and it is on his *sworn* testimony that I based the statements made. If Dr. Blackburn will read Dr. Worcester's book, and then read his evidence in the Guiteau trial, and contrast the views, he will alter his opinion as to Dr. W.'s ability and honesty. I am not an "allopath," nor do I know that such a sect exists. I am not opposed to the therapeutic views other than as opposed to my idea of what is rational. I object to sectarian designations in science, but am not even a bigot in this respect. Dishonesty in the statement of science is what I am opposed to, and from the extract cited you can judge how just my criticism of Dr Worcester was. Very sincerely,

JAS. G. KIERNAN.

---

I, Alex. Bruce, being duly sworn, on oath say that I was nurse of the male surgical ward, Cook County Infirmary, November 1, 1882, when Joseph C. McMullen was admitted with the left leg amputated in the middle third. That I was present at the first and all subsequent dressings of the amputated member up to the time of the death of the said J. C. McMullen, and that at the first dressings no ends of ligatures were outside of the stump; their absence was remarked upon at that time, and that the first ligatures seen was after the stump had sloughed, when a large bundle and several short ligatures were seen in the stump, and that both bones of the leg protruded beyond the end of the stump when the second amputation was made by Drs. Morgan and Bennett, of the Cook County Hospital. ALEX. BRUCE.

Mr. Alex. Bruce, personally known to me, has been duly sworn to the facts stated and subscribed to above.

[NOTARIAL SEAL.] G. J. WOLF,
Dunning, April 18, 1884. Notary Public.

---

CHICAGO, April 7, 1884.

I, Wm. E. Morgan, being duly sworn, on my oath do say: That on November 16, 1882, at Cook County Infirmary, State of Illinois, during the temporary absence of Dr. A. W. Hagenbach, and with the assistance of Dr. Bennett, then interne of Cook County Hospital, I re-amputated a sloughing stump on the left leg of one J. C. McMullen, and that the following is a verbatim copy of a note written by me in the hospital records concerning said stump; also that the description is correct, and was verified by me by autopsy of the amputated part:

"Stump sloughed and opened up, revealing a large bunch of silken ligatures left in the stump. A second operation was deemed advisable, so the operation was performed on the 16th, a re-amputation being made one and a half inches higher than the first."

I do not wish to enter into any professional discussion, but I remember distinctly the condition of the stump, and make this affidavit simply from the facts then existing, regardless of whom the statement may hit. There were many ligature ends, some short and some long, wadded into a bundle, having been some time previously sewed up, *bagged*, if I may use the expression, within the stump, and the ends of both tibia and fibula protruded from the stump. WM. E. MORGAN, M.D.

3160 State Street, Chicago, Ill.

Subscribed and sworn to before me this seventh day of April, A. D., 1884.

[NOTARIAL SEAL.] D. MCDONALD, Notary Public.

---

STATE OF MINNESOTA, }

County of Hennepin. }

Dr. E. R. Bennett, being duly sworn, and on his oath deposes and says, that on November 16, 1882, at the Cook County Infirmary, in the county of Cook, State of Illinois, during the temporary absence of Dr. A. W. Hagenbach, I assisted William E. Morgan, then interne of the Cook County Hospital, in re-amputating a "sloughing stump" on the left leg of one J. C. McMullen, and that the following is a correct description of the stump before re-amputation: Stump sloughing, flaps ulcerated and shortened, with end of tibia and fibula protruding. On opening up the stump, I distinctly remember finding a large bunch of silken ligatures matted together which were left in the stump. A second operation being deemed advisable, a re-amputation was performed upon the 16th day of November, 1882.

DR. E. R. BENNETT.

Subscribed and sworn to before me this 16th day of April, 1884.

[NOTARIAL SEAL.] JOHN F. HAYES, Notary Public.
Hennepin County, Minn.

---

GENTLEMEN:—In your April number, pages 439 and 442, appears a criticism by one J. G. Kiernan, on "Insanity and its Treatment," by Samuel Worcester, M.D., concerning which I wish to say a few words. I do not object to any honest, plain criticism of any medical work; for so much chaff is written that we have to wade through oceans of it to gather one little grain of truth; but I do earnestly protest against personal abuse, such as appears in the article mentioned. Dr. Worcester is called a "homœopathic renegade," which would imply that he is a traitor to his homœopathic principles or faith. I suppose the author meant he was a traitor to allopathic principles. *This is not true*, and only illustrates the bitter malignity with which the allopathic school has always pursued those who dared to differ with them. That it was meant to be personal is proven upon page 442, where Dr. W. is charged with "dishonest therapeutic views," and it seems to me that such remarks concerning a gentleman of Dr. S. Worcester's standing could not be written by a decent man, or published in a respectable medical journal.

G. E. BLACKBURN.

No. 2819 Cottage Grove Avenue, April 15, 1884.

